# Wind-Induced Vibration Monitoring of High-Mast Illumination Poles Using Wireless Smart Sensors

**DOI:** 10.3390/s24082506

**Published:** 2024-04-14

**Authors:** Mona Shaheen, Jian Li, Caroline Bennett, William Collins

**Affiliations:** Department of Civil, Environmental and Architectural Engineering, The University of Kansas, Lawrence, KS 66045, USA; mshaheen@ku.edu (M.S.); crb@ku.edu (C.B.); william.collins@ku.edu (W.C.)

**Keywords:** wind-induced vibration, buffeting, vortex shedding, high-mast illumination poles, crosswind, along wind, wireless smart sensors

## Abstract

This paper describes the use of wireless smart sensors for examining the underlying mechanism for the wind-induced vibration of high-mast illumination pole (HMIP) structures. HMIPs are tall, slender structures with low inherent damping. Video recordings of multiple HMIPs showed considerable vibrations of these HMIPs under wind loading in the state of Kansas. The HMIPs experienced cyclic large-amplitude displacements at the top, which can produce high-stress demand and lead to fatigue cracking at the bottom of the pole. In this study, the natural frequencies of the HMIP were assessed using pluck tests and finite element modeling, and the recorded vibration frequencies were obtained through computer vision-based video analysis. Meanwhile, a 30.48 m tall HMIP with three LED luminaires made of galvanized steel located in Wakeeney, Kansas, was selected for long-term vibration monitoring using wireless smart sensors to investigate the underlying mechanism for the excessive wind-induced vibrations. Data analysis with the long-term monitoring data indicates that while vortex-induced vibration occurs frequently at relatively low amplitude, buffeting-induced vibration was the leading cause of the excessive vibrations of the monitored HMIP. The findings provide crucial information to guide the design of vibration mitigation strategies for these HMIP structures.

## 1. Introduction

High-mast illumination poles (HMIPs) are tall (typically 24.38 to 45.72 m), slender structures. They are usually found in open areas near highways or rest areas where a high level of illumination is required. These structures are known for having low inherent damping. As a result, they are susceptible to excessive vibrations due to wind loading through various vibration mechanisms such as vortex shedding, buffeting, and galloping, which can lead to premature fatigue cracking in the HMIPs.

Vortex shedding occurs due to negative pressure caused by alternating wind vortices on the side of the structure, causing the structure to oscillate in the direction perpendicular to the wind [1]. When the frequency of vortex-induced vibration (VIV) approaches the structure’s natural frequency, a “lock-in” phenomenon occurs [2]. VIV occurs within a specific range of wind speeds, known as the lock-in velocity [1], for a particular mode of the structure. For HMIPs, the lock-in phenomenon typically happens at higher modes, such as the second or third mode [3]. On the other hand, buffeting is excited by wind turbulence caused by wind gusts, resulting in vibration response with participation from multiple modes, typically dominated by the first mode [4]. The amplitude of this type of vibration is known to increase with the increase in wind speed, causing the structure to experience large displacements [5]. The vulnerability of slender structures, such as light poles, telecommunication towers, chimneys, and other slender structures, to vibrations caused by crosswind and torsional buffeting exceeds that of susceptibility to along-wind buffeting [6]. Typically, structures are not designed to withstand dynamic loads due to crosswind and torsional buffeting, as designs are limited to static analysis according to the maximum crosswind and torsion loads and unit dynamic coefficient [6]. Slender structures exhibiting low natural frequencies, e.g., less than 1 Hz, rough surface, odd aerodynamic shape, and low damping, are susceptible to buffeting-induced vibration [7,8]. Such vibration becomes significant under high mean wind speed and high-intensity turbulence [7,8]. Galloping-induced vibration is featured by unstable aerodynamic vibration with large amplitude, usually occurring in the crosswind direction [5]. Galloping is influenced by the lift and drag coefficients of the structure, as well as the wind attack angle. Because the lift and drag coefficients for a circular section are constant, galloping vibration is unlikely to occur in HMIPs with circular sections unless rain or snow adheres to the structure’s surface, changing its circumferential characteristics [9].

Previous studies on light pole structures distinguished the mechanism behind excessive wind vibrations by identifying the natural frequencies and modes involved in the vibration and determining the alignment of the light pole’s dominant vibration direction with the wind direction. For instance, Lloyd et al. [10] monitored four tapered HMIPs in Wyoming over two years, with heights ranging from 30.47 to 36.58 m. The study found that vibrations due to second-mode lock-in, which occurred in the crosswind direction, were more frequent than vibrations caused by along-wind buffeting. In addition, three large amplitude events associated with first-mode lock-in were observed in the direction perpendicular to the wind. Ice was present during two of the three events, yet there were instances with ice buildup and comparable wind speeds that did not lead to first-mode lock-in. Their study could not determine why some wind events resulted in lock-in while others did not, but it proposed that the lock-in might be attributed to the alignment of wind characteristics with the light pole’s resonance frequency. Puckett and Ahearn [3] conducted a three-month monitoring of four tapered 36.6 m HMIPs. The collected acceleration data revealed crosswind vibrations associated with third-mode lock-in due to vortex shedding at critical wind speeds between 4.47 and 8.49 m/s. Zuo and Letchford [5] investigated the vibrations of a tapered 12.19 m cantilevered light pole. By converting the acquired acceleration data into displacement and considering the wind direction relative to the light pole vibration, the presence of buffeting and VIV were both identified. In particular, buffeting-induced vibrations were more prevalent than those caused by vortex shedding. Buffeting-induced vibrations exhibited unsteady vibration amplitudes, which intensified when wind speed increased and occurred in diverse wind directions relative to the pole vibration. On the other hand, VIV was dominated by the second mode under specific wind speed ranges, with the pole vibration perpendicular to the wind direction. 

In the United States, HMIPs are designed according to the “AASHTO Standard Specifications for Structural Supports for Highway Signs, Luminaires and Traffic Signals” [11] to withstand different loads, including wind loads. However, the wind loading described by these specifications is mainly based on wind tunnel testing, which may not fully reveal the wind excitation mechanism [5]. Studies showed that long-term wind-induced vibrations on these light poles can create cumulative fatigue damage, cracks, and potential failure in high-stress areas [12]. For instance, in November 2003, a 42.67 m tall high-mast tower collapsed along the highway I-29 in Sioux City, Iowa, due to high wind speed [13]. When the high-mast tower collapsed, the wind speed was 16.54 m/s, with a reported maximum wind speed of 25.03 m/s on that day. Connor and Hodgson [13] monitored several high-mast towers in Iowa and found that buffeting vibrations due to natural wind gusts, which occurred at the first natural frequency, caused the highest stress cycles. Meanwhile, the study found that vortex shedding caused second-mode lock-in, which occurred at a specific range of wind speed ranges between 1.34 and 4.47 m/s. The measured stress due to vortex shedding was approximately 13.8 MPa, which is a low-stress range; however, the potential for stress accumulation exists due to the number of cycles caused by VIV. Another study by Caracoglia and Jones [9] was motivated by the collapse of 134 light poles in February 2003 at seven different interchanges on highway I-74, US-150, and US-34 in Galesburg, Illinois. These light poles were installed between 1997 and 2000. The collapsed light poles were 13.70 m high and equipped with a rod-in-canister damper installed at two-thirds of the light pole height from the base and tuned to prevent second-mode vibrations due to vortex shedding. The large oscillations caused failure in the breakaway support, the most commonly reported type of failure, or fracture caused by cracks initiating at the pole’s handhole. The study excluded along-wind buffeting, fatigue cracks due to long-term exposure to turbulence buffeting, and VIV as potential causes for the collapse of light poles since the failure modes and existing weather conditions did not align with these mechanisms. For example, when the light poles collapsed, the wind was steady with no turbulence, and the wind speed was 22 m/s. In addition, there was heavy snow with temperatures below zero degrees Celsius when the light poles collapsed. Instead, Caracoglia and Jones [9] deduced that the significant vibration amplitudes were triggered by uncommon events that combined wind and icy precipitation, ultimately leading to the failure. In April 2004, two HMIP structures failed in Denver and collapsed at the E-470 intersection near the Denver International Airport [14]. The collapse was due to crack initiation and propagation under high-stress cycles due to wind. Therefore, Goode and van de Lindt [14] developed a design procedure based on a reliability index to improve the fatigue life of HMIP structures subjected to wind loads. More studies examined the aerodynamic behavior and the underlying causes of wind-induced vibrations in light poles [3,5,15].

Previous studies showed that HMIPs are susceptible to excessive wind vibrations caused by vortex shedding and buffeting, which can result in catastrophic consequences if these light poles fail and collapse. While vortex shedding occurs at a specific wind range and frequency, buffeting occurs over a broader range of frequencies and increases with increasing wind speed. The research objective of this study is to determine the primary mechanisms responsible for the excessive wind-induced vibrations on HMIPs in Kansas. Therefore, long-term field monitoring was carried out, followed by a detailed analysis of the collected data in correlation to the wind speed and direction. The findings of this study provide critical information to guide the design of vibration mitigation strategies. The remainder of the paper is structured as follows. Section 2 presents a preliminary investigation through the video analysis of an HMIP excessively vibrating due to wind. Section 3 provides a detailed description of the long-term field monitoring program. Section 4 discusses the dynamic characteristics of the monitored HMIP obtained from a pluck test. Section 5 discusses the data analysis. Section 6 presents the key findings of the study.

## 2. Preliminary Investigation

**Figure 1:**
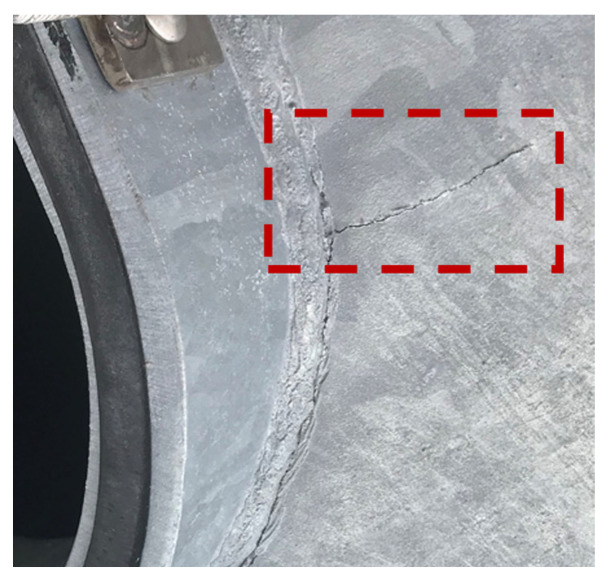
A premature crack (shown in the red box) near the handhole of an HMIP in West Kansas (Credit: KDOT).

**Figure 2:**
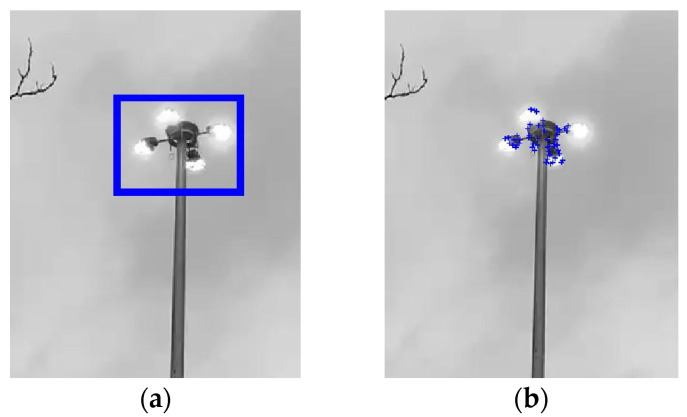
(**a**) Region of interest for displacement tracking. (**b**) Detected feature points used in the tracking.

**Figure 3:**
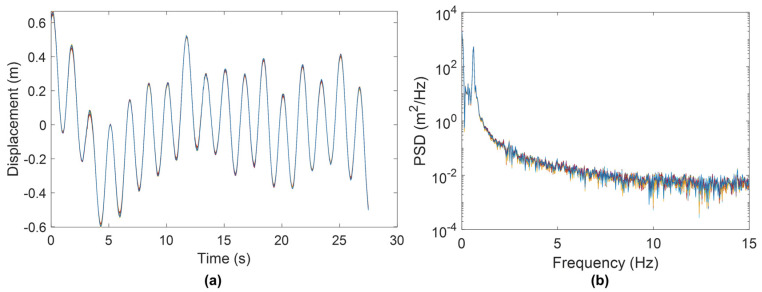
(**a**) Displacement time histories of feature points from the top of the HMIP extracted from the video. (**b**) Power spectral density (PSD) of the tracked displacements showing a dominant frequency of 0.61 Hz.

The Kansas Department of Transportation (KDOT) discovered premature cracks near the handhole of several newly installed HMIPs in western Kansas [16], as shown in Figure 1. In addition, KDOT recorded several videos of the HMIPs experiencing large cyclic displacements at the top of the HMIP due to wind loading. Hence, a preliminary study was conducted on the recorded videos to investigate the nature of the excessive vibrations. The videos were analyzed using the Kanade–Lucas–Tomasi (KLT) feature tracking approach to extract the maximum displacement and vibration frequencies of the structure. First, the region of interest around the light assembly was selected to track the displacement at the top of the HMIP, where the maximum displacement occurs, as shown in Figure 2a. The sampling frequency of the recorded video was 29.97 Hz, which was used to track the displacement time history using the KLT feature point tracker [17] for the detected feature points in Figure 2b. Subsequently, the tracked displacement was utilized to estimate the natural frequency of the HMIP. Results indicated that the average peak-to-peak displacement was approximately 0.60 m, as shown in Figure 3a. In addition, the power spectral density (PSD) of the tracked displacement showed that a mode with a natural frequency of 0.61 Hz dominated the vibration, as illustrated in Figure 3b. The preliminary conclusion will be compared with the pluck test results, FE modeling, and wind-induced vibration data. Following the observation of significant vibrations and the preliminary video-based modal analysis, FE modeling, monitoring, and data analysis were conducted to further investigate the causes of the HMIP’s vibrations. 

## 3. Long-Term Field Monitoring

Long-term vibration monitoring was conducted on a 30.47 m tall HMIP with three LED luminaires in western Kansas. It should be noted that the HMIP selected for monitoring is not identical to the ones seen in the videos, but they share similar characteristics such as material properties, height, slenderness ratio, structural and luminaire details, and similar natural frequencies. A wireless smart sensor network was deployed on the selected HMIP over a three-month period to collect acceleration responses and wind data, including wind velocity and direction. Extensive data analysis was performed to characterize the nature of wind-induced HMIP vibrations to uncover the reason behind the vibrations with excessive amplitudes. Alongside long-term monitoring, pluck tests were also performed to determine the natural frequencies and the inherent damping ratio of the HMIP.

### 3.1. Location and Geometry of the Selected HMIP

**Figure 4:**
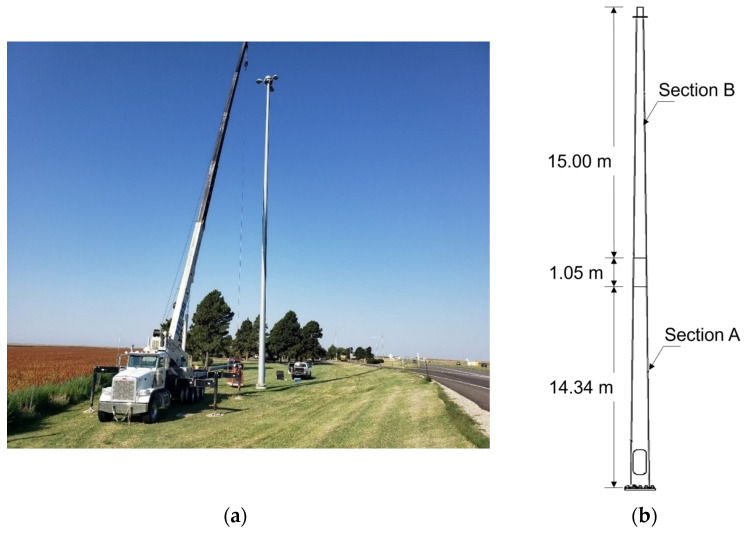
(**a**) The selected HMIP located in Wakeeney, Kansas, for long-term monitoring. (**b**) Schematic of the HMIP.

**Figure 5:**
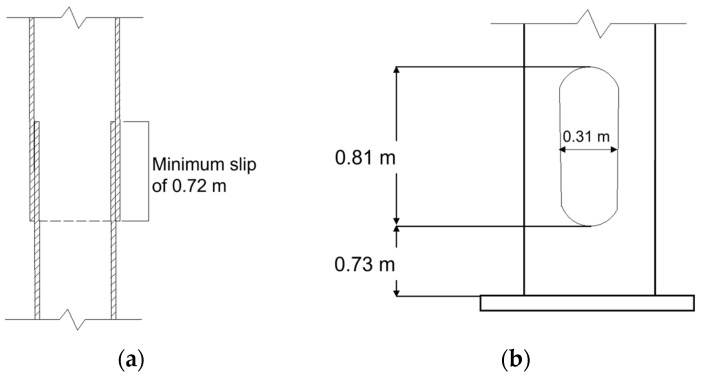
(**a**) Slip joint details. (**b**) Handhole details.

The selected 30.47 m tall HMIP is located in an open highway rest area in Wakeeney, Kansas, as shown in Figure 4a. The HMIP has a 0.33-degree tapered circular section made of galvanized steel. As illustrated in Figure 4b, it consists of two sections with different heights, with dimensions summarized in Table 1. According to the design, a minimum of 0.72 m overlap is required by KDOT, as illustrated in Figure 5a. However, the manufacturer did not define the exact overlap between sections A and B; later, it was estimated to be 1.05 m based on the height at which the outer diameter of part A matches the base inner diameter of part B. 

A light assembly is located at the top of the pole, which has a mass of 184.16 kg and consists of three LED light fixtures and a lowering device. The HMIP has a handhole that is 0.31 m wide and 0.81 m tall and is located 0.73 m above the base plate, as depicted in Figure 5b. The handhole allows access to operate the lowering device and carry out maintenance of the HMIP. 

### 3.2. Wireless Smart Sensor Network

**Figure 6:**
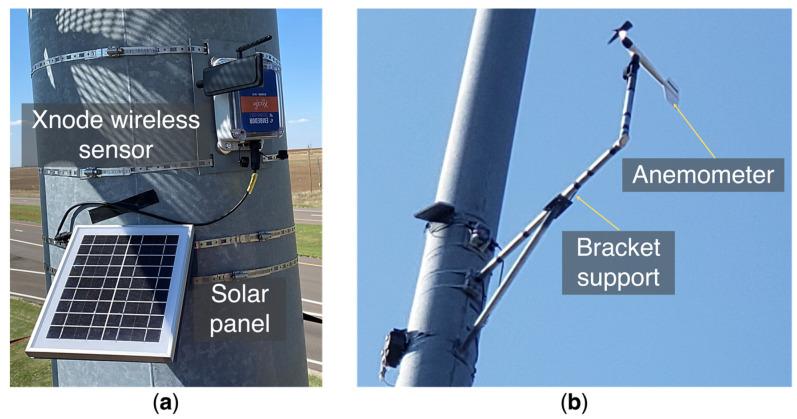
(**a**) Xnode wireless sensor and solar panel. (**b**) Anemometer and the bracket support.

A wireless smart sensor network was designed for long-term field monitoring using Xnodes from Embedor Technologies [18], as shown in Figure 6a. The wireless smart sensor network consists of three remote sensor nodes and one gateway sensor node; each of them is equipped with triaxial accelerometers. One of the remote sensor nodes was connected to an M. Young propeller vane anemometer (Model 05103V), as illustrated in Figure 6b, through a breakout box to measure the wind speed and direction. The sensor nodes are powered by rechargeable batteries and solar panels, and the anemometer is powered using a 12 V battery connected to a solar panel. The sensitivity of the wind speed output of the anemometer is 50 mV per m/s, and the sensitivity of the wind direction output is 13.9 mV per degree [19]. The sensor nodes spend most of the time in deep sleep mode to conserve energy and can be triggered to wake up immediately for data collection through an onboard low-power accelerometer, which constantly monitors the ambient vibration level. A programmable acceleration threshold is stored in each sensor node for triggering data collection for the sensor nodes under a large vibration. Since each sensor node is triggered independently, the data collected by the sensor nodes are not synchronized. The gateway sensor node communicates with the other three remote sensor nodes to collect their data and upload it to the cloud using a 4G cellular network. The cloud server and database can be accessed from a website where data can be visualized and downloaded. The website has an encrypted account for login access to ensure security. Also, it provides the interface to remotely change parameters of the sensor networks, such as the triggering threshold.

**Figure 7:**
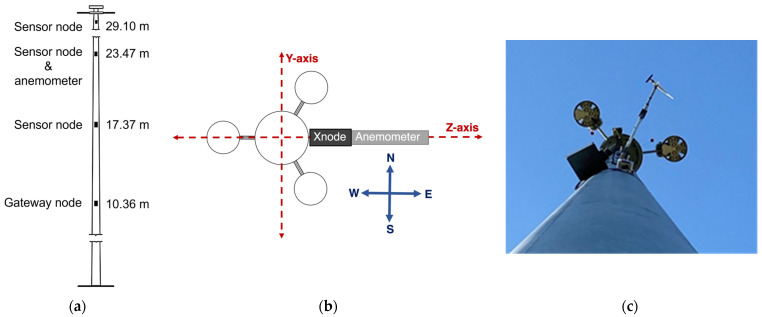
(**a**) Locations of the deployed wireless sensors along the HMIP; (**b**) Illustration of sensor orientations; (**c**) Alignment of the sensors and the luminaries.

The sensors were installed on the HMIP at different heights, as shown in Figure 7a, determined based on the HMIP’s mode shapes obtained from an FE model, such that the sensor captured vibrations from multiple modes. The cellular gateway node was placed at the lowest elevation where the vibrations are minimized, so it will be triggered less often to ensure reliable communication with the remote sensors and upload the data to the cloud server. The anemometer was installed at the height of 23.47 m and connected to the Xnode located at the same height. A 1.22 m long bracket support was used to support the anemometer to reduce the impact of wind turbulence for reliable wind measurements. The recommended support length is six times the HMIP diameter to minimize upstream and downstream flow distortions [20]. However, for practical reasons, the support length adopted in the bracket was 3.2 times the HMIP diameter. The anemometer data were compared with a nearby weather station to ensure consistency despite the shorter bracket length. In this investigation, understanding the relationship between the direction of vibration and the wind direction is critical to determine the main reason behind the excessive wind vibration. The orientation of the sensors with the light assembly is shown in Figure 7c. Figure 7b shows the placement of sensor nodes in the eastern direction. The *Z*-axis of the sensor nodes is aligned in the east–west direction and with the 0-degree angle of the anemometer.

## 4. Pluck Test

Pluck tests were performed after instrumenting the HMIP to determine the natural frequencies and inherent damping of the first mode of the monitored HMIP. Estimating the damping ratio for higher modes from the pluck test data was challenging due to the non-stationary nature of the collected data. The damping ratios were overestimated when an exponential window was applied to the acceleration time history to reduce the response amplitude by 1%. Therefore, the study looked at the inherent damping ratio of the first mode.

**Figure 8:**
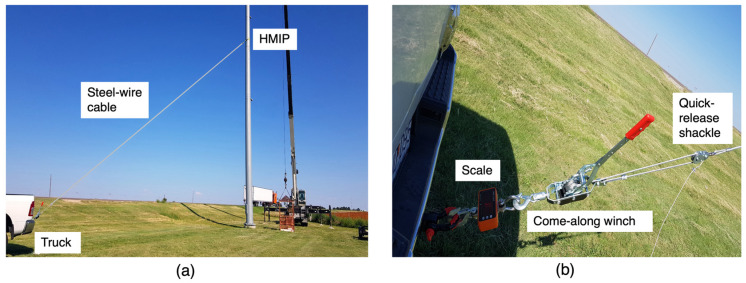
(**a**) Illustration of the pluck test. (**b**) Tools used in the pluck test.

**Figure 9:**
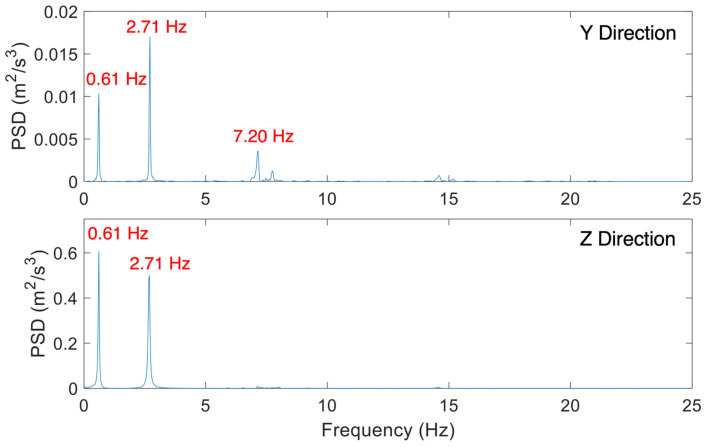
Natural frequencies identified from the pluck test.

The test was conducted by attaching a steel wire cable to the HMIP at a height of 15.24 m from the base, then connecting it to a quick-release shackle attached to a pickup truck equipped with a come-along winch and a load scale, as shown in Figure 8. An initial displacement was applied to the HMIP by pulling the cable using the come-along winch and then releasing it with the quick-release shackle to let the HMIP vibrate freely. In the meantime, the sensor network was triggered to collect the acceleration response under free vibration. Figure 9 displays the natural frequencies of the HMIP obtained from the pluck test. The first mode frequency was 0.61 Hz, the second mode was 2.71 Hz, and the third mode was 7.20 Hz.

The HMIP damping ratio was estimated using the measured free vibration response from the pluck test. First, the measured acceleration data obtained from the sensor at the height of 29.10 m were filtered using a low-pass elliptic filter with a cut-off frequency of 1.8 Hz to remove frequencies above the first natural frequency. Then, from the filtered free decay response, the damping ratio can be estimated using the logarithmic decrement (δ) method based on two peak amplitudes separated by *N* cycles, in which δ is given by

(1) $$\delta =ln\left(\frac{U_{P}}{U_{Q}}\right)=\zeta \omega_{n}T_{d}N=\frac{2\pi N\zeta }{\sqrt{1-\zeta^{2}}}$$

where $U_{P}$ is the peak amplitude of the first cycle and $U_{Q}$ is the peak magnitude of the (*N* + 1)th cycle. $T_{d}$ is the damped natural period, which is equal to $\frac{2\pi }{\omega_{d}}$ = $\frac{2\pi }{\omega_{n}\sqrt{1-\zeta^{2}}}$. For a small damping ratio, $\sqrt{1-\zeta^{2}}\approx 1$. Thus, the damping ratio can be approximated using the following equation:

(2) $$\zeta =\frac{1}{2\pi N}ln\left(\frac{U_{P}}{U_{Q}}\right)$$

**Figure 10:**
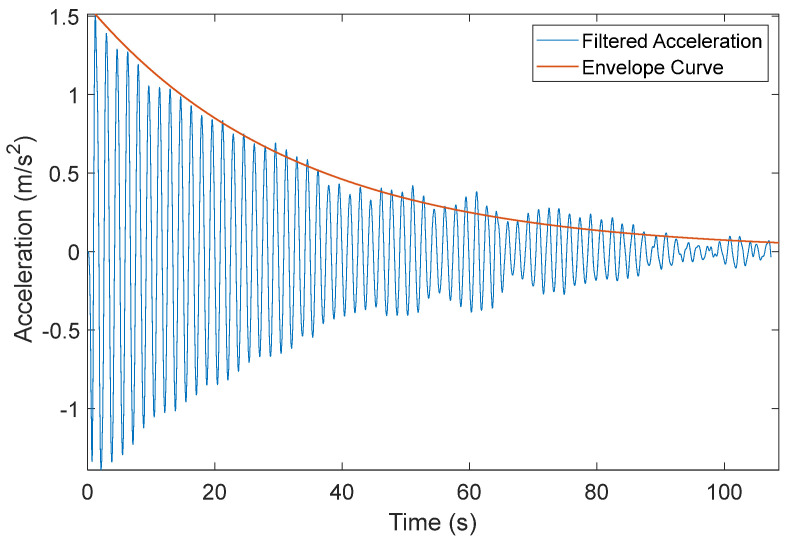
Low-pass filtered free vibration response from the pluck test at a height of 29.1 m.

Subsequently, the first mode damping ratio was estimated to be around 0.8% using the logarithmic decrement method given in Equation (2). Figure 10 verifies the estimated damping ratio by showing the filtered free vibration response from the pluck test and its envelope curve associated with a 0.8% damping ratio.

## 5. Data from Long-Term Vibration Monitoring Results

**Figure 11:**
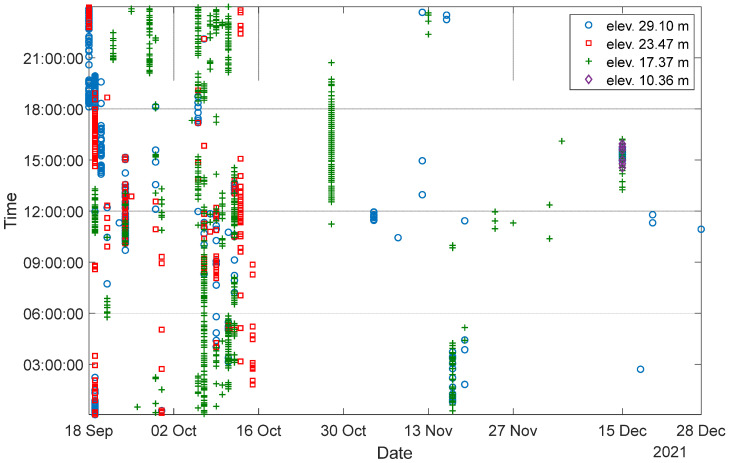
Overview of data collected over the three-month monitoring period.

The HMIP was monitored for three months, from 18 September 2021 to 28 December 2021. Figure 11 summarizes the collected data in terms of date and time. During the monitoring period, 1239 datasets were collected. The data were sampled at 50 Hz. The triggering threshold for the accelerometer was initially set at 70 mg for the first month to assess the vibration level of the HMIP under wind load, and then it was raised to 300 mg to limit data collection for only significant wind events. As a result, as shown in Figure 11, more datasets were collected during the first month, and only significant wind events were recorded during the other two months. The gap in the collected data after 16 October 2021 is attributed to the sensors losing battery charge due to some excessive wind vibrations that repeatedly triggered the sensors on cloudy days, during which time the solar panels could not recharge the batteries. 

### 5.1. Identified and Analytical Modal Properties

The natural frequencies of the HMIP were identified using the PSD functions obtained from the collected acceleration data, which were validated by a preliminary FE model based on the detailed drawings and design documents of the HMIP. The first three natural frequencies in the y-direction were determined to be 0.61 Hz, 2.73 Hz, and 7.48 Hz, respectively. Additionally, the first three natural frequencies in the z-direction were identified as 0.60 Hz, 2.68 Hz, and 7.32 Hz, respectively. The slight difference in natural frequencies in the y and z directions could be attributed to signal processing uncertainties and minor asymmetries in the structure. 

**Figure 12:**
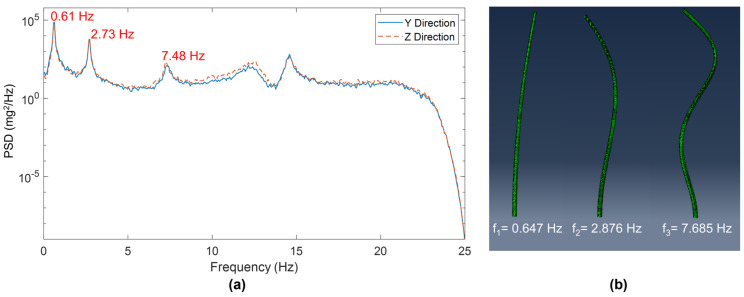
(**a**) Natural frequencies from wind-induced vibration. (**b**) Natural frequencies and mode shapes from the FE model.

An FE model of the HMIP was created based on the design drawing using an eight-node linear brick element (C3D8) in Abaqus CAE, version 6.24 [21]. The HMIP was modeled as two tapered segments with a lumped mass at the top, corresponding to the light assembly and lowering device. Figure 12b illustrates the first three analytical mode shapes of the modeled HMIP, and the corresponding natural frequencies are 0.65 Hz, 2.88 Hz, and 7.69 Hz, respectively. Table 2 summarizes the analytical natural frequencies obtained from the FE model and those identified from the wind-induced vibration measurements. As mentioned, the mode shapes were not obtained from the field monitoring because the sensors were triggered independently; hence, the measured acceleration responses were not synchronized.

### 5.2. Data Analysis

This study aims to assess the cause of excessive wind vibration of the HMIP based on the monitored acceleration and wind (speed and direction) data collected by the wireless smart sensor network. The acceleration data were converted to displacement using a time-domain Finite Impulse Response (FIR) filter to avoid low-frequency drift when the double integration method is used. A detailed description of the adopted method can be found in [23,24]. 

To identify the underlying mechanisms behind the HMIP wind-induced vibrations, we investigated the modes that contribute to the HMIP vibration and how the vibration aligns with the wind direction. The relationship between wind direction and movement of the HMIP was assessed by creating a wind rose diagram for each dataset, which is illustrated later in this section. The wind rose diagram consists of the magnitude and direction of the recorded displacement in conjunction with the corresponding wind speed and direction for each dataset obtained from the anemometer. Two types of wind-induced vibrations were identified during the data analysis, including VIV and buffeting, which are discussed further in this section.

#### 5.2.1. Vortex-Induced Vibration (VIV)

VIV is characterized by vibrations in the crosswind direction, typically with the HMIP response being locked in at the second or third mode at a critical wind velocity [1,3,10,25]. In this study, the monitored HMIP experienced VIV in the second mode. The critical wind velocity depends on the Strouhal number ($S_{t}$) and can be expressed as $U=\frac{f_{s}D}{S_{t}}$, where $S_{t}$ equals 0.18 for a circular section, according to AASHTO [11]. Here, $f_{s}$ represents the second mode frequency, and D denotes the diameter of the HMIP. Hence, the critical wind velocity for the second-mode lock-in ranges between 4.47 and 7.60 m/s. 

**Figure 13:**
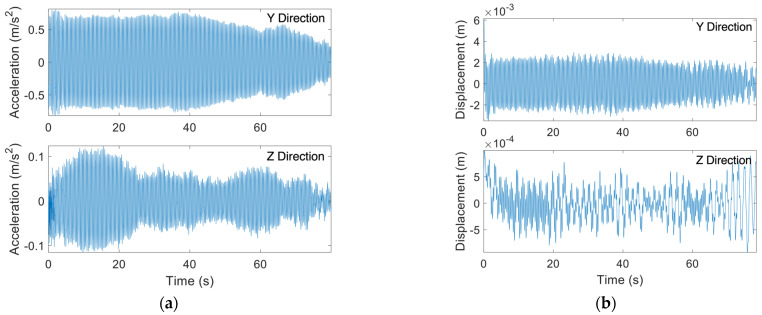

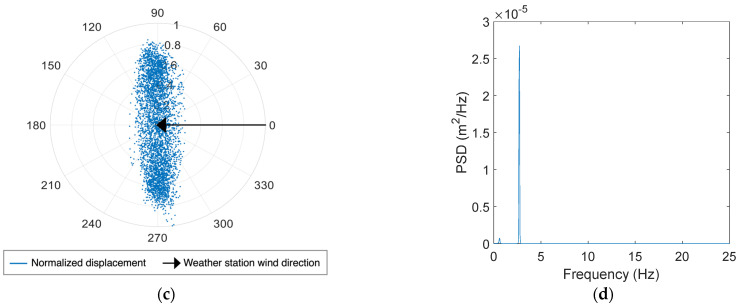
(**a**) Collected acceleration time histories for VIV; (**b**) Displacement time histories obtained from the collected acceleration data for VIV; (**c**) wind rose for VIV showing crosswind vibration; (**d**) PSD of the total displacement responses showing lock-in during VIV.

Figure 13a illustrates acceleration time histories for the HMIP under VIV as recorded by the sensor installed at an elevation of 17.37 m. The corresponding displacement for the same wind event is shown in Figure 13b. The nearest weather station [26] reported a wind speed of 4.47 m/s, with the wind blowing from the east. Unfortunately, the sensor connected to the anemometer was not triggered when the dataset was collected. However, the anemometer readings from other wind events were consistent with the weather station. Therefore, the data analysis in this section is based on the wind information provided by the weather station. The displacement in the *Y*-axis shown in Figure 13b indicates that the HMIP was engaged in VIV during the wind event. The wind was blowing from the eastern direction, aligning with the *Z*-axis of the Xnode. It can be seen that the *Z*-axis displacement was negligible compared to the *Y*-axis. The normalized wind rose for the data in Figure 13c shows that the HMIP vibrated in the north–south direction while the wind direction was from the east, resulting in a crosswind vibration. The total PSD obtained by summing the PSDs of the *Y*-axis and *Z*-axis is illustrated in Figure 13d. The PSD curve demonstrates a second-mode lock-in, where the second mode dominates the vibration. Given that the wind speed reported by the weather station was 4.47 m/s, falling within the critical wind velocity range, the HMIP experienced VIV in the crosswind direction and was dominated by the second mode. Although VIV was reported to occur more frequently than buffeting in HMIPs and other tall, slender structures [1,3,10,26], during the monitoring period, VIV was observed to have lower amplitudes and happen less often than buffeting. 

#### 5.2.2. Buffeting

Buffeting occurs due to the turbulence component in the wind, causing the irregular motion of a structure. This is commonly observed in slender structures with low frequencies [27]. The structural response due to buffeting is directly proportional to the wind intensity and tends to be higher at higher wind speeds [8,27]. The first mode usually dominates buffeting-induced vibration, while other modes can also contribute to its vibration response [4,5,9]. 

**Figure 14:**
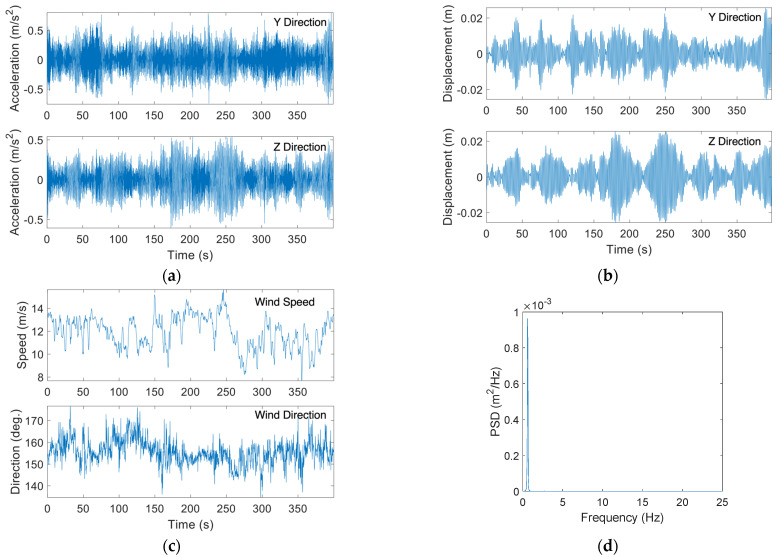
(**a**) Collected acceleration time histories for along-wind buffeting-induced vibration; (**b**) Displacement time histories obtained from the collected acceleration data for along-wind buffeting; (**c**) Collected wind speed and wind direction during along-wind buffeting excitation; (**d**) PSD of the total displacement responses due to buffeting.

Figure 14a,b shows typical acceleration and displacement time histories due to buffeting from the monitoring campaign. The wind speed and direction for the same dataset are demonstrated in Figure 14c. It can be noted that wind speed fluctuated between a maximum of 15.65 m/s and a minimum of 7.82 m/s, while the wind direction was consistent and around 155 degrees. The wind turbulence intensity, which is defined as the standard deviation of the wind speed ($u^{\prime }$) over the mean wind speed (U) [27], is high and around 12%. The displacement amplitude in Figure 14b fluctuates with the wind speed, where the maximum displacement the pole experienced during the wind event was 0.0254 m. The PSD curve corresponding to the displacement data in Figure 14d shows that the first mode dominated the vibration of the HMIP. Overall, vibrations due to buffeting exhibited amplitude fluctuation depending on the wind speed variation and were dominated by the first mode.

**Figure 15:**
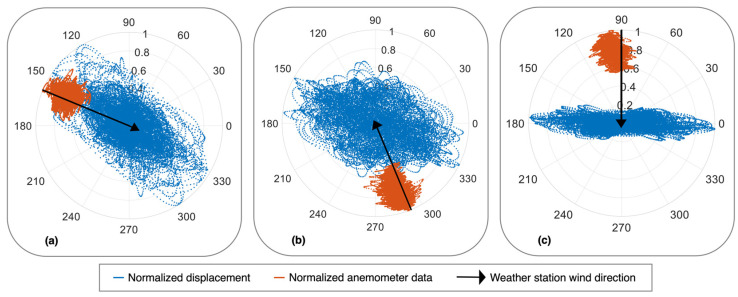
(**a**–**c**): Wind roses for buffeting-induced vibration showing different angles between the HMIP vibration and the wind direction.

The normalized wind rose diagrams for the HMIP in Figure 15 illustrate the direction of the HMIP’s movement and the wind direction for different wind events due to buffeting. Figure 15a illustrates the normalized magnitude and direction of displacement in Figure 14b and the normalized anemometer data from Figure 14c. During the wind event, the HMIP vibrates in the same direction as the wind due to buffeting. On the other hand, Figure 15b shows another wind event where buffeting was the primary vibration source; however, we can see the HMIP is moving at an oblique angle to the wind direction. Furthermore, Figure 15c illustrates the HMIP vibrating perpendicular to the direction of the wind. 

**Figure 16:**
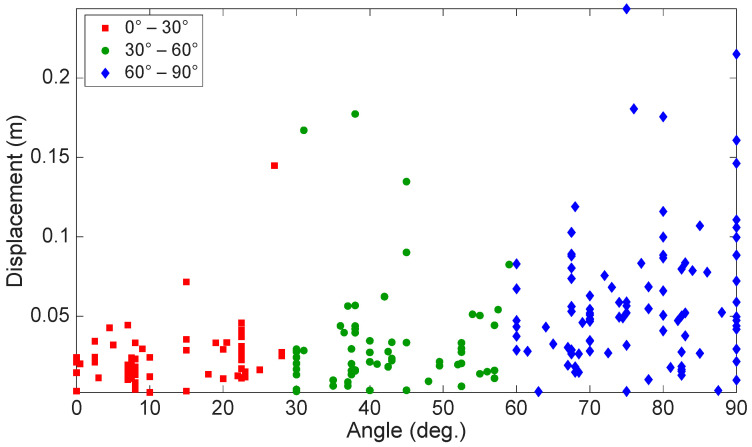
Displacement magnitude and the acute angle between the wind direction and the HMIP vibration from the accelerometer located at 29.10 m elevation.

While most studies indicate that buffeting occurs primarily in the along-wind direction [1,3,9], only a few discuss buffeting occurring in the crosswind direction. For example, Zuo and Letchford [5] showed that buffeting occurred over a broad range of wind directions, with the highest vibration amplitudes happening when buffeting was in the crosswind direction and dominated by the first mode. Phares et al. [7] found that HMIP is susceptible to high-amplitude oscillations at significantly higher wind speeds due to buffeting occurring in the crosswind direction in the presence of self-excited forces. During data analysis, we found that buffeting occurred over a wide range of angles, while the crosswind vibrations have the highest magnitudes, as shown in Figure 16, which presents the displacement magnitude and the acute angle between the direction of the wind and the buffeting-induced HMIP vibration. The angles were grouped into intervals of 30 degrees, representing along-wind, oblique wind, and crosswind directions. It can be noted that the vibration occurred over a wide range of angles from 0 to 90 degrees, with higher amplitudes in the crosswind direction. A few exceptions to the data in the along-wind and oblique-wind directions were during a Derecho storm, which will be discussed later in this section. 

**Figure 17:**
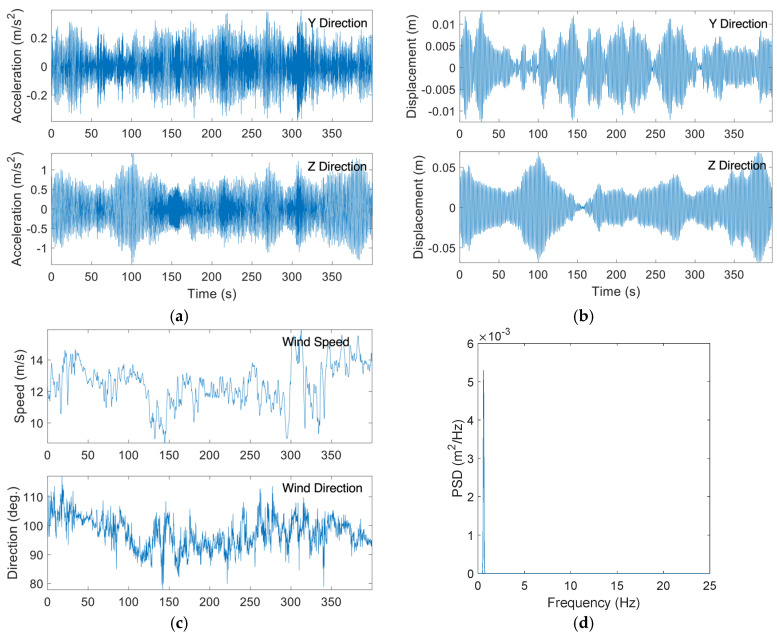
(**a**) Collected acceleration time histories for crosswind buffeting-induced vibration; (**b**) Displacement time histories obtained from the collected acceleration data for crosswind buffeting; (**c**) Collected wind speed and wind direction for crosswind buffeting; (**d**) PSD of the total displacement responses due to crosswind buffeting.

The acceleration and displacement time history in Figure 17a and Figure 17b, respectively, are for the same dataset shown in Figure 15c with the crosswind vibration. The displacement exhibited a fluctuating behavior that varies as the wind speed changes. Figure 17c shows that the wind speed fluctuated between 8.94 and 15.65 m/s while the wind direction remained constant at approximately 100 degrees. The wind turbulence intensity is high, around 11%. Upon examining the PSD derived from the displacement data, it was observed that the HMIP’s vibration was predominantly governed by the first mode, as illustrated in Figure 17d.

Crosswind vibration with a single frequency dominating the vibration can be attributed to VIV, buffeting, or galloping. Regarding VIV due to wind crossing the main pole body, the critical velocity due to vortex shedding and first-mode lock-in ranges between 0.98 and 1.65 m/s, which is 10 times less than the one experienced by the HMIP; therefore, VIV associated with the main pole structure was excluded as the source of this excessive vibration in the crosswind direction. In addition, VIV can also be caused by the light assembly on the top of the HMIP. Junge [28] performed a series of computational fluid dynamic (CFD) analyses for the light assembly under various wind velocities and angles. The study indicated that VIV could occur close to the first mode under a specific wind speed and angle. However, the data we collected indicate the HMIP experienced crosswind vibration under various wind directions, including north, north-northwest, west-southwest, and south, indicating that the light assembly did not contribute to the first mode lock-in. The 11% turbulence intensity suggests that the vibration could not be due to galloping, which is caused by quasi-steady forces. Additionally, no ice or snow was present to create an asymmetric shape of the HMIP when the data were collected. Consequently, galloping was excluded as a potential reason for this excessive vibration. Thus, it was concluded that buffeting is the primary cause of this crosswind vibration. This type of buffeting-induced vibration exhibited a higher vibration magnitude than others triggered by buffeting. For instance, the displacement amplitudes for the crosswind buffeting in Figure 17b are almost twice the magnitude of those in Figure 14b, which were associated with crosswind buffeting.

#### 5.2.3. Summary of Data Analysis

**Figure 18:**
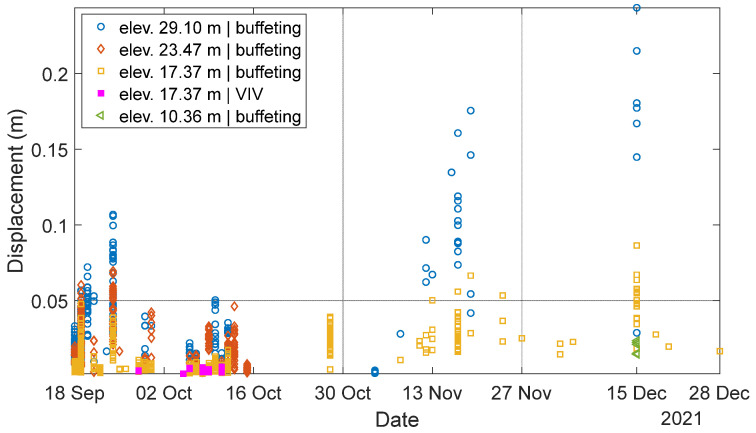
Overview of the wind-induced vibration and displacement amplitude from the long-term monitoring.

Data analysis revealed that the HMIP experienced two types of wind-induced vibrations: VIV and buffeting. Figure 18 shows the magnitude of displacement for the vibrations induced by wind due to these two types of vibrations, in which buffeting-induced vibrations are labeled by hollow markers, while VIVs are labeled by solid markers. It can be seen that buffeting occurred more frequently than VIV. In addition, the displacement amplitudes from buffeting are significantly higher than ones from VIV, indicating that buffeting-induced vibration is likely the leading cause of the observed excessive vibrations. Therefore, in terms of both occurrences and magnitudes, buffeting represents a much bigger contributor to the overall vibration experienced by the HMIP.

As shown in Figure 18, the highest displacement magnitude was recorded during the long-term monitoring period on 15 December 2021, when a Derecho storm occurred. This type of storm is known for high wind speeds and large wind gusts. According to the nearby weather station, the maximum recorded wind speed on that day was 25.93 m/s, and the maximum wind gust reached 37.55 m/s [26]. Unfortunately, the data from the wireless smart sensor connected to the anemometer was unavailable that day. Therefore, the excessive wind vibrations during the Derecho storm were investigated according to the wind data from the weather station and the data collected by the other three sensors. 

**Figure 19:**
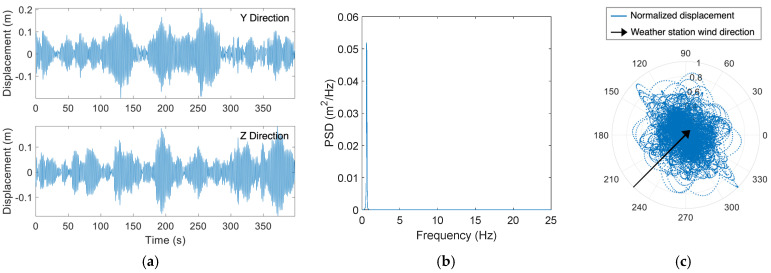
(**a**) Displacement time histories captured during the Derecho storm; (**b**) the total PSD; (**c**) normalized wind rose for total displacement during the Derecho storm.

**Figure 20:**
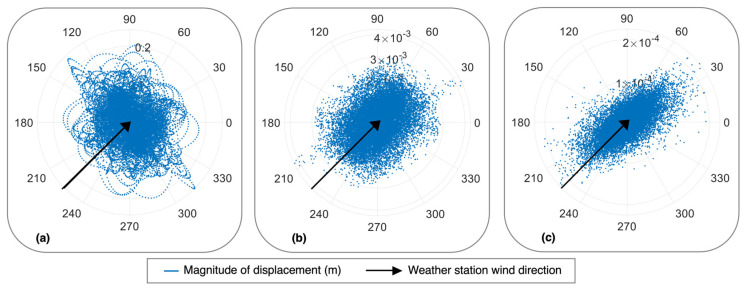
Wind roses for (**a**) the first mode component, (**b**) the second mode component, (**c**) the third mode component. Note that the displacements are not normalized.

The displacement time history derived from the accelerometer located at the height of 29.10 m during the storm is shown in Figure 19a. The displacement data exhibited fluctuation and reached large amplitudes. The maximum displacement the HMIP experienced was about 0.24 m, which is the closest displacement magnitude to the 0.30 m captured in the video from KDOT. Such large displacement can induce high stress and potentially cause fatigue cracking near the base of the HMIP over time [28]. Figure 19c shows the wind direction compared to the normalized magnitude and direction of the HMIP displacement. According to the weather station, the wind blew from the southwest (SW) direction. Therefore, the wind rose plot indicates that the vibration primarily occurred in the crosswind direction. Additionally, Figure 19c shows the HMIP moving in different directions, but it is mainly dominated by crosswind vibration. To further evaluate the contribution from each mode, an 8th-order bandpass elliptic filter was applied to the measured acceleration. Subsequently, the filtered acceleration data were converted into displacement to assess the modal contributions to the HMIP vibration during the storm. Figure 20 represents the displacement magnitude and direction associated with the first three modes and the wind direction. The results indicate that the first mode contributed the most, with a displacement magnitude of 0.24 m. In contrast, the effects of the second and third modes are significantly lower than that of the first mode. Hence, the excessive wind vibration experienced during the Derecho storm exhibited crosswind buffeting characteristics due to its fluctuating displacement, large vibration amplitude, and first-mode dominance in the HMIP vibration.

## 6. Conclusions

This study examined the mechanism of the excessive wind-induced vibrations on a 30.48 m tall HMIP in Wakeeney, Kansas. First, the video analysis of multiple videos captured by KDOT was carried out using the KLT feature tracking method to extract the HMIP’s maximum displacement and vibration frequencies. In addition, a three-month field monitoring was conducted to collect acceleration and wind data to investigate the main reason behind the excessive vibrations. 

The video analysis indicated that the vibration is dominated by a 0.61 Hz frequency and a maximum displacement of 0.30 m, which was confirmed to be the first-mode frequency through the pluck tests and FE analysis. Furthermore, the long-term field monitoring revealed that the HMIP experienced two types of wind-induced vibrations: buffeting and vortex shedding. Vibrations caused by buffeting are excited by the turbulence component of wind load, resulting in vibration over a wide range of angles. The study found that the vibration magnitude when buffeting occurred in the crosswind direction was higher compared to buffeting-induced vibrations that occurred in other directions. Additionally, the vibration response from buffeting was governed by the first mode. On the other hand, vortex shedding excites the structure only in the crosswind direction at critical wind speeds. The critical velocities for VIV range between 4.47 and 7.60 m/s for the second-mode lock-in. However, VIV occurred less frequently and exhibited lower magnitudes than buffeting.

The maximum recorded displacement was 0.24 m, measured at the top of the HMIP, which occurred during the Derecho storm. This value is the closest to the 0.3 m displacement extracted from the video during the preliminary investigation. Additionally, the first mode dominates the vibration from both the video recording and the Derecho storm. Therefore, the data analysis indicated that the excessive wind vibration experienced by the HMIP is attributed to crosswind buffeting. Such excessive vibrations can generate high-stress demand at the bottom of the structure, initiating and propagating fatigue cracks such as fatigue cracking around the handhole and potentially resulting in the failure of the structure if left unaddressed. The findings from this study suggest that future research for vibration mitigation should focus on increasing the damping level of HMIPs by implementing structural dampers instead of aerodynamic dampers since aerodynamic dampers change the aerodynamic properties of the system to disrupt the formation of vortices. However, the study indicates that VIV is not the main cause of excessive vibrations. As a result, using aerodynamic dampers will not help reduce unwanted vibrations.

## Figures and Tables

**Table 1:** Dimensions of the HMIP cross-sections.

	Bottom Diameter (m)	Top Diameter (m)	Length (m)	Thickness (cm)
Section A	0.65	0.47	15.48	0.64
Section B	0.49	0.30	16.06	0.48

**Table 2:** Analytical and identified natural frequencies of the HMIP.

	First Mode	Second Mode	Third Mode
FE model	0.65 Hz	2.88 Hz	7.69 Hz
Wind-induced vibration (Y direction)	0.61 Hz	2.73 Hz	7.48 Hz
Wind-induced vibration (Z direction)	0.60 Hz	2.68 Hz	7.32 Hz

Table 2 from Shaheen et al. [22].

## Data Availability

Data are available upon request.

## References

[1] Chang B., Phares B.M., Sarkar P.P., Wipf T.J. (2009). Development of a procedure for fatigue design of slender support structures subjected to wind-induced vibration. Transp. Res. Rec..

[2] Wu T., Kareem A. (2012). An overview of vortex-induced vibration (VIV) of bridge decks. Front. Struct. Civ. Eng..

[3] Puckett J., Ahearn E.B. (2010). Reduction of Wind-Induced Vibrations in High-Mast Light Poles.

[4] Chen W., Zhang Q., Li H., Hu H. (2015). An experimental investigation on vortex induced vibration of a flexible inclined cable under a shear flow. J. Fluids Struct..

[5] Zuo D., Letchford C.J. (2008). Investigation of Wind-Induced Highway Lighting Pole Vibration Using Full-Scale Measurement.

[6] Solari G. (2018). Gust buffeting of slender structures and structural elements: Simplified formulas for design calculations and code provisions. J. Struct. Eng..

[7] Phares B.M., Sarkar P.P., Wipf T.J., Chang B. (2007). Development of Fatigue Design Procedures for Slender, Tapered Support Structures for Highway Signs, Luminaries, and Traffic Signals Subjected to Wind-Induced Excitation from Vortex Shedding and Buffeting.

[8] Chang B. (2011). Aerodynamic Parameters on a Multisided Cylinder for Fatigue Design. Wind Tunnels and Experimental Fluid Dynamics Researc.

[9] Caracoglia L., Jones N.P. (2007). Numerical and experimental study of vibration mitigation for highway light poles. Eng. Struct..

[10] Lloyd J.B., Connor R.J., Sherman R.J. (2020). Field Testing and Long-Term Monitoring of Selected High-Mast Lighting Towers.

[11] American Association of State Highway and Transportation Officials (AASHTO) (2015). Standard Specifications for Structural Supports for Highway Signs, Luminaires and Traffic Signals.

[12] Tsai L.W., Alipour A. (2020). Assessment of fatigue life and reliability of high-mast luminaire structures. J. Constr. Steel Res..

[13] Connor R., Hodgson I. (2006). Field instrumentation and testing of high-mast lighting towers in the state of Iowa. Draft Final Report.

[14] Goode J.S., van de Lindt J.W. (2007). Development of a semiprescriptive selection procedure for reliability-based fatigue design of high-mast lighting structural supports. J. Perform. Constr. Facil..

[15] Foley C.M. (2004). Structural Analysis of Sign Bridge Structures and Luminaire Supports.

[16] Yount T., Yu D., Bennett C., Collins W., Li J. (2024). Investigation of High Mast Illumination Pole Handhole Cracking.

[17] Almarshad A. (2020). Structural Health Monitoring Strategies Using Traditional Sensors and Computer Vision, in Civil Engineering. Ph.D. Thesis.

[18] Fu Y., Hoang T.A., Mechitov K., Kim J.R., Zhang D., Spencer B.F. (2018). Sudden Event Monitoring of Civil Infrastructure Using Demand-Based Wireless Smart Sensors. Sensors.

[19] R. M. Young Company (2000). Wind Monitor With Voltage Outputs Model 05103V: Instructions.

[20] Barthelmie R.J., Wang H., Doubrawa P., Pryor S. (2016). Best Practice for Measuring Wind Speeds and Turbulence Offshore through In-Situ and Remote Sensing Technologies.

[21] ABAQUS (2021). ABAQUS/CAE 6.24 User’s Manual.

[22] Shaheen M., Li J., Taher S., Bennett C., Collins W. Wind-induced vibration monitoring of high mast illumination poles. Proceedings of the Sensors and Smart Structures Technologies for Civil, Mechanical, and Aerospace Systems 2022.

[23] Almarshad A., Li J., Lepage A. Drift Estimation of Tall Building Structures under Non-stationary Wind Loading through Sensor Data Fusion. Proceedings of the 9th International Conference on Structural Health Monitoring of Intelligent Infrastructure (ISHMII-9).

[24] Park J.-W., Sim S.H., Jung H.-J. (2013). Displacement Estimation Using Multimetric Data Fusion. IEEE/ASME Trans. Mechatron..

[25] Han Y., Zhou X., Wang L., Cai C., Yan H., Hu P. (2021). Experimental investigation of the vortex-induced vibration of tapered light poles. J. Wind Eng. Ind. Aerodyn..

[26] Hays Regional Station (2021). Hays, KS Weather History.

[27] Simiu E., Scanlan R.H. (1996). Wind Effects on Structures: Fundamentals and Applications to Design.

[28] Junge B.A. (2022). Computational Fluid Dynamics Investigation into High Mast Illumination Poles: Influence of Light Fixture Type.

